# Specific cultivation-independent enumeration of viable cells in probiotic products using a combination of fluorescence *in situ* hybridization and flow cytometry

**DOI:** 10.3389/fmicb.2024.1410709

**Published:** 2024-06-12

**Authors:** Laura Snaidr, Peter Mühlhahn, Claudia Beimfohr, Christian Kreuzer, Carolin Richly, Jiri Snaidr

**Affiliations:** Vermicon AG, Hallbergmoos, Germany

**Keywords:** Flow-FISH, enumeration, specific count, viability count, multi-species blend, FISH, probiotics, plate count

## Abstract

This study introduces an optimized integration of flow cytometry and fluorescence *in situ* hybridization (Flow-FISH) as an approach for the specific enumeration of gram-positive bacteria in probiotic products, overcoming the limitations of conventional methods. The enhanced Flow-FISH technique synergizes the rapid and automated capabilities of flow cytometry with the high specificity of FISH, facilitating the differentiation of viable cells at the species level within probiotic blends. By analyzing lyophilized samples of *Lacticaseibacillus rhamnosus*, *Lactiplantibacillus plantarum*, and *Bifidobacterium animalis* subsp. *lactis*, and a commercial product, the study highlights the optimized Flow-FISH protocol’s advantages, including reduced hybridization times to 1.5 h and elimination of centrifugation steps. Comparative evaluations with the widely accepted enumeration methods plate count and Live/Dead (L/D) staining were conducted. The study revealed that Flow-FISH produces higher viable cell counts than plate count, thereby challenging the traditional “gold standard” by highlighting its predisposition to underestimate actual viable cell numbers. Against L/D staining, Flow-FISH achieved comparable results, which, despite the different foundational premises of each technique, confirms the accuracy and reliability of our method. In conclusion, the optimized Flow-FISH protocol represents a significant leap forward in probiotic research and quality control. This method provides a rapid, robust, and highly specific alternative for the enumeration of probiotic bacteria, surpassing traditional methodologies. Its ability to enable a more detailed and reliable analysis of probiotic products paves the way for precise quality control and research insights, underscoring its potential to improve the field significantly.

## 1 Introduction

Over the past few decades, research interest in microbiomes has increased significantly. It is now widely accepted that the human gut microbiome is not only crucial for proper digestion but also plays a vital role in a functioning immune system and is implicated in the development of various diseases such as irritable bowel syndrome and diarrhea (Menees and Chey, 2018; Li et al., 2021). Evidence also suggests its influence on neurodevelopmental and neurodegenerative diseases like Autism Spectrum Disorder, Alzheimer’s, and Parkinson’s Disease (Cryan et al., 2019; Loh et al., 2024).

This has generated considerable interest in the composition of a healthy microbiome and how it might be effectively influenced. Although understanding the microbiome has proven to be a complex endeavor, as the microbiomes of healthy people are not homogeneous in their composition (Eckburg et al., 2005; Arumugam et al., 2011; Pasolli et al., 2019), it has been discovered that certain types of bacteria, known as probiotics, positively impact human health when consumed alive and in sufficient quantities (Kerry et al., 2018; Ranjha et al., 2021; Latif et al., 2023).

The World Health Organization defines probiotics as “live microorganisms that, when administered in adequate amounts, confer a health benefit on the host” (Hill et al., 2014). This definition raises the question about methods for accurately quantifying not just the cells but specifically the viable cells.

Davis (2014) proposed that not only probiotic cells capable of reproducing but also all cells that are metabolically active and/or have an intact membrane should be considered alive. Hence, one of the major challenges posed by the plate count method is the so-called viable but non-culturable (VBNC) state. Those cells can enter a dormant state, during which they remain metabolically active but do not replicate, thus losing their ability to be cultured. However, they might effectively recover in the human gut, as it provides the appropriate environment (Lahtinen et al., 2006b; Davis, 2014; Fiore et al., 2020).

Since probiotic cells are often exposed to a stressful environment during industrial production as well as during storage, VBNC cells are frequently found in probiotic products (Mills et al., 2011; Wendel, 2022).

The most commonly utilized bacteria in probiotics are lactic acid bacteria, specifically *Lactobacillus* and *Bifidobacterium* species (Williams, 2010). Jackson et al. (2002) confirmed among other studies that the culture-based method does not always provide accurate insights into lactic acid bacteria (Moreno et al., 2006; Pereira et al., 2023).

An alternative method of great interest in the field of probiotics is flow cytometry (Adan et al., 2017; Pane et al., 2018; Sielatycka et al., 2021; Boyte et al., 2023; Pereira et al., 2023; Tracey et al., 2023) due to its rapid and automatic results. Staining the cells with fluorescent dyes prior to measurement can provide valuable information. The applications of fluorescent dyes in this context are diverse, including the determination of nucleic acid content, enzyme activities, and apoptotic cells, as well as the identification of cell surface receptors and diverse cell populations (Adan et al., 2017).

Additionally, DNA intercalating fluorescent dyes, which can penetrate the cell differently depending on the integrity of the membrane, are commonly used for probiotic cell labeling to determine their viability state, thus making flow cytometry suitable for quantifying the number of live cells in a sample. However, this method has severe limitations when applied to multiple species blends, which are often found in practice, as it is a non-specific method (ISO 19344:2015, Tracey et al., 2023).

Consequently, the combination of flow cytometry with a technique that permits the specific identification of microorganisms is not merely advantageous but essential for advancing the capabilities of flow cytometric analysis. Fluorescence *in situ* hybridization (FISH) is a suitable technology for this purpose as it facilitates the differentiation not only between viable and non-viable cells but also among distinct species.

The principle of FISH is based on the phylogeny of microorganisms (Woese, 1987) and utilizes fluorescently labeled oligonucleotide probes that target specific sites at the ribosomal RNA (rRNA) of the microorganisms. It enables the precise detection of viable microbial populations ranging from broad taxonomic groups to individual species and involves fixing microbial cells to stabilize and permeabilize them, followed by hybridization with fluorescently labeled oligonucleotide probes and subsequent analysis via epifluorescence microscopy (Amann et al., 1990a, 1995; Wagner et al., 1993; Snaidr et al., 1997). FISH has proven to be a powerful tool for the simultaneous visualization and characterization of multiple bacterial populations in the same sample (Amann et al., 1996; Lukumbuzya et al., 2019).

For probiotics, the FISH method has been successfully applied multiple times to identify and quantify various probiotic species in fecal and lyophilized samples (Langendijk et al., 1995; Rinne et al., 2005; Bezirtzoglou et al., 2011; Pasulka et al., 2021).

Although the classical FISH method offers some significant advantages, such as rapid results, specificity, and differentiation between live and dead cells, the standardization of microscopic evaluation is challenging due to its dependence on the performer. Consequently, the combination of FISH with methods enabling automated, objective, and thus standardized quantification is required. The first combination of flow cytometry and FISH, the so-called Flow-FISH dates back about 30 years (Amann et al., 1990a; Wallner et al., 1993; Snaidr et al., 1999).

Since then, both FISH and flow cytometry techniques have significantly advanced, and Flow-FISH has been successfully demonstrated for fecal microorganisms (Rigottier-Gois et al., 2003; Rochet et al., 2004; Vaahtovuo et al., 2005; Dinoto et al., 2006; Collado and Sanz, 2007; Cleusix et al., 2010).

However, the protocols required extended hybridization times of more than 10 hours, which is far from a rapid method. Moreover, they require several centrifugation steps which might lead to cell loss and by this negatively influence quantitative data.

In this study, we present an optimized and advanced combination of flow cytometry and FISH, demonstrating its suitable application, especially in the field of probiotics. The objective of the study was to demonstrate and validate the efficacy of the Flow-FISH method in accurately and specifically enumerating gram-positive bacteria species in both single and mixed blends of probiotics. For this purpose, we analyzed different lyophilized probiotic samples consisting of *Lacticaseibacillus rhamnosus*, *Lactiplantibacillus plantarum*, and *Bifidobacterium animalis* subsp. *lactis*, and a commercial product, comparing the outcomes of this advanced Flow-FISH methodology with those of established techniques such as Live/Dead (L/D) measurement via flow cytometry and plate count analysis.

## 2 Materials and methods

### 2.1 Sample preparation

Lyophilized strains of *Lacticaseibacillus rhamnosus* SP1 (*L. rhamnosus*), *Lactiplantibacillus plantarum* LP-115 (*L. plantarum* LP-115) and 14D (*L. plantarum* 14D), and *Bifidobacterium animalis* subsp. *lactis* BLC1 (*B. lactis*), as well as a mix of these strains, were rehydrated according to ISO 19344:2015. In detail, 100 mg of each lyophilized strain, as well as from the self-mixed sample, was diluted at a 1:20 w/v ratio using a 0.1% peptone salt solution. Rehydration was done by shaking the samples at 100 rpm for 60 min at room temperature. For analyzing the commercially available product “IberoBiotics Pro” (Lot number: 69974, expiry date: February 2025; Bayer Vital GmbH, Leverkusen, Germany), the entire capsule content was transferred into a 50 mL tube and rehydrated in 50 mL of 0.1% peptone salt solution. The sample was shaken at 100 rpm for 60 min at room temperature. Immediately after rehydration, the samples were further processed.

### 2.2 Quantification via plate count

For the cultivation of the strictly anaerobic *B. lactis*, an anaerobic environment was established utilizing an airtight container in combination with anaerobic packs (Sigma Aldrich, Darmstadt, Germany) to ensure the absence of oxygen. *B. lactis* was cultured on DSM Medium 58 agar at 37°C. The microaerophilic *L. rhamnosus* and *L. plantarum* 14D were cultured on MRS agar at 37°C in a suitable atmosphere, utilizing microaerobic packs (BioMérieux SA, Marcy-l’Étoile, France). Each sample underwent a serial 10-fold dilution. Adequate dilutions were plated and incubated for 48 to 96 h. Grown colonies were counted and results reported as colony forming units (CFUs) per gram.

### 2.3 Analysis by Live/Dead staining

The LIVE/DEAD BacLight Bacterial ViabilityKit (Thermo Fisher Scientific Inc., Waltham, Massachusetts) was used to differentiate between live and dead cells by differential staining following the ISO 19344:2015 standard. The two dyes utilized were SYTO 9 and propidium iodide (PI), which differ in their spectral characteristics and ability to penetrate intact cells. SYTO 9, a green fluorescent nucleic acid stain, enters all cells regardless of membrane integrity. In contrast, PI is selective in entering cells with compromised membranes only, i.e., dead or damaged cells, thereby causing a reduction in SYTO 9 fluorescence within these cells. The extent of PI penetration and subsequent fluorescence reduction is dependent on the level of membrane damage, leading to either a very low signal of green fluorescence in presumed dead cells or a partial decrease in green fluorescence in damaged cells. In summary, this staining technique enables differentiation between living, damaged, and dead cells.

To examine the cells, 1.5 μL of each dye was added to a tube containing 997 μL of PBS buffer and mixed well. 990 μL of this dying solution was mixed with 10 μL of diluted rehydrated sample and incubated for 15 min at room temperature in the dark. The sample was then immediately measured on a Cytek Northern Lights flow cytometer (Cytek Bioscience Inc., Fremont, CA, United States). Results were reported as total fluorescence units (TFUs) and active fluorescence units (AFUs) per gram.

### 2.4 Analysis by Flow-FISH

Rehydrated sample was mixed in a 1:1 v/v ratio with lysozyme (Sigma Aldrich, Darmstadt, Germany), at a species-specific optimized concentration of 400,000 Units/mL for *L. rhamnosus* or 833,000 Units/mL for *L. plantarum* 14D and *B. lactis*, and incubated for 30 min (*L. rhamnosus* and *L. plantarum* 14D) or 5 min (*B. lactis*), at 40°C. For hybridization, 40 μL of a double strength hybridization buffer (40% formamide, 40 mM Tris HCl, 1800 mM NaCl, and 0.02% SDS) with 200 ng/μL of the specific, fluorescently labeled oligonucleotide probe was added to 40 μL of the lysozyme-treated sample. Hybridization was carried out in a heating block at 40°C for 90 min. In experiments involving a multi-species blend, the hybridization buffer was prepared with 200 ng/μL of each required fluorescently labeled oligonucleotide probe. Blend was treated with 400,000 Units/mL of lysozyme for 15 min at 40°C. For analyzing the commercial product, the rehydrated product was processed with the same lysozyme treatment as the lyophilized strains for *L. rhamnosus* and *B. lactis*, and with 83,000 units/mL for 1 min at 40°C for *Lactobacillus acidophilus*.

In this study, fluorescent dye was consistently attached to the 5′ end of each oligonucleotide probe. In single species experiments, the respective specific probe was labeled with the fluorescent dye 6-Carboxyfluorescein (6-FAM). To detect multiple species within a blend, the EUB338 probe (Amann et al., 1990b) was used for total bacterial count, and labeled with 6-FAM. For specific detection, *L. rhamnosus* probe was labeled with DY-415, *L. plantarum* specific probe was labeled with Cy3, and *B. lactis* specific probe was labeled with DY-631, allowing for the differentiation and quantification of these species in mixed cultures based on their unique 16S rRNA signatures (Table 1).

**Table 1:** List of labeled oligonucleotides used in this study.

Probes	Sequences 5`-3`	Target organisms	16S/23S rRNA	Lysozyme treatment for Flow-FISH	Probe sequence references
EUB338	GCT GCC TCC CGT AGG AGT	All bacteria	16S	Dependent on the target organism	Amann et al. (1990b)
EUB338_Quencher	ACT CCT ACG GGA GGC AGC	–	–	–	This study
S-S-Lrham-1586-a-A-23	AGC ACC TTT CAA TAA TCA GAA CT	*Lacticaseibacillus rhamnosus*	16S	400.000 Units/mL, 30 min, 40°C	Goldberg et al. (2000)
S-S-Lrham-1586-a-A-23_Quencher	AGT TCT GAT TAT TGA AAG GTG CT	–	–	–	This study
Lbpla462	CCG TCA ATA CCT GAA CAG TTA C	*Lactiplantibacillus plantarum*	16S	833.000 Units/mL, 30 min, 40°C	This study
Lbpla462_Quencher	GTA ACT GTT CAG GTA TTG ACG G	–		–	This study
Biflac65	CAA GCT GCC AGG GAT CCC GT	*Bifidobacterium animalis* subsp. *lactis*	16S	833.000 Units/mL, 5 min, 40°C	This study
Biflac65_Quencher	ACG GGA TCC CTG GCA GCT TG	–		–	This study
Lbaci1872	TCG AAC CTT CGC TTT CGC	*Lactobacillus acidophilus*	23S	83.000 Units/mL, 1 min, 40°C	This study
Lbaci1872_Quencher	GCG AAA GCG AAG GTT CGA	–		–	This study

To mitigate non-specific signals arising from potential unspecific oligonucleotide binding, 40 μL of triple strength washing buffer was added post-hybridization. Washing buffer (60 mM Tris HCl, 645 mM NaCl, and 15 mM EDTA) contained 300 ng/μL oligonucleotide quencher probes complementary to the specific probes used for hybridization, linked with a corresponding quencher at the 3′ end. Washing was carried out in a heating block at 40°C for 15 min. For 6-FAM, BMN-Q535 was used as a quencher, for DY-415, BMN-Q460, and for Cy3 and DY-631, BMN-Q620 (Table 1).

**Figure 1:**
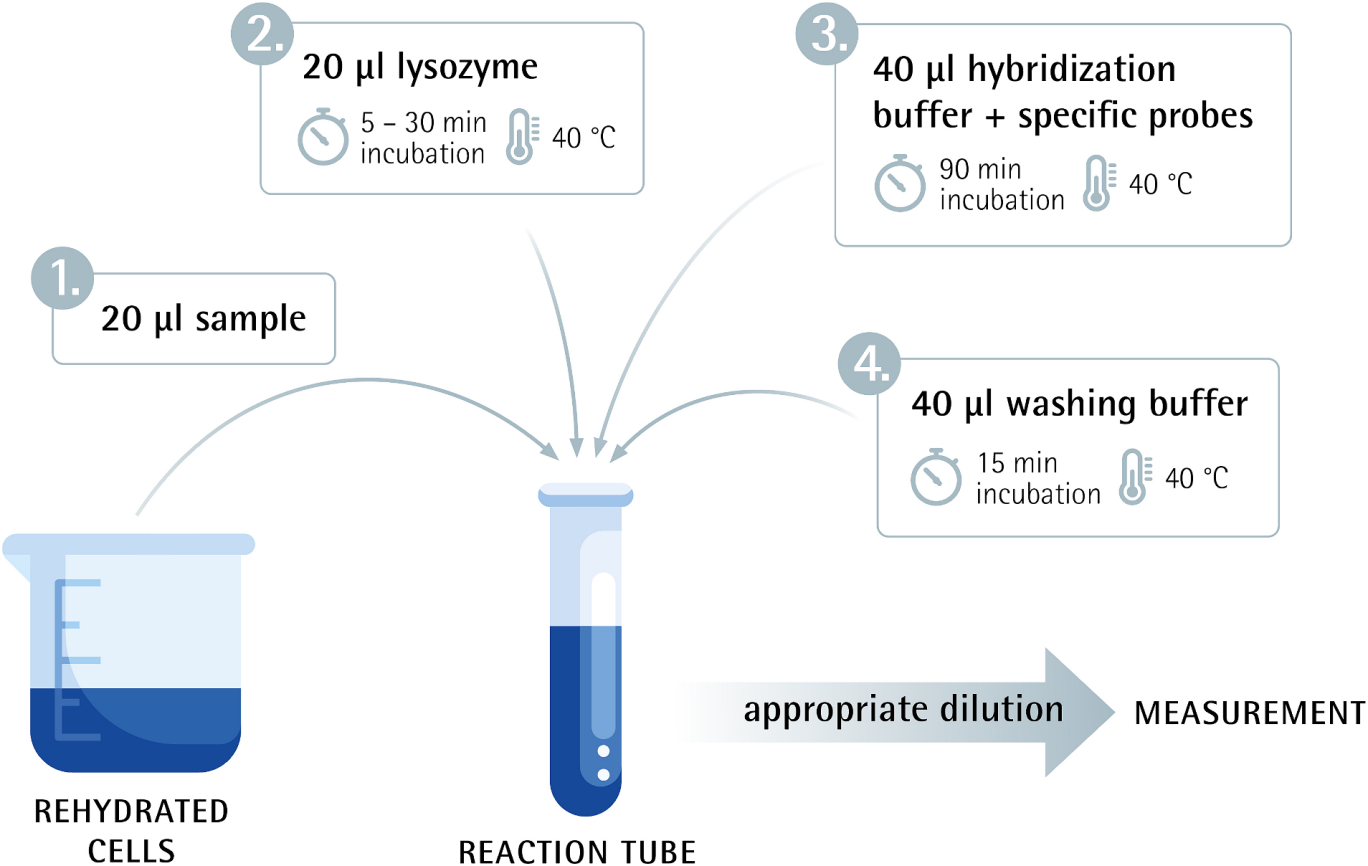
Flow-FISH method overview combining flow cytometry and fluorescence *in situ* hybridization.

To evaluate the linearity of the Flow-FISH method, a rehydrated sample was serially diluted three times in 10-fold steps. The undiluted sample and the four subsequent dilutions were then processed according to the protocol. Before being analyzed with the Cytek flow cytometer, the samples were further diluted to achieve an optimal event rate for measurement. Results were measured in “viable cells”/g (Figure 1).

### 2.5 Flow cytometer measurement

Flow cytometry measurements for this study were conducted using a Cytek Northern Lights flow cytometer (Cytek Biosciences Inc., Fremont, CA, United States), equipped with a three-laser system. The cytometer’s lasers configuration, featuring 405 nm (100 mW), 488 nm (50 mW), and 640 nm (80 mW) lasers. This setup facilitated the measurement of forward scatter (FSC) and side scatter (SSC) alongside fluorescence detection across a wide emission spectrum (420–829 nm) without the necessity for filter changes. Data analysis was performed using SpectroFlo^®^ Software Version 3.2.1 (Cytek Biosciences, Inc.).

For L/D staining analysis, cells were discriminated from the background by gating on a positive SYTO 9 fluorescence signal. Discrimination between living, dead and damaged was achieved by correlation plots between SYTO 9 and PI intensity. Living cells were selected in an area with high SYTO 9 fluorescence, whereas dead cells were selected in a region of high PI fluorescence and lower SYTO 9 fluorescence. Damaged cells exhibit intermediate levels of both SYTO 9 and PI fluorescence. Flow rate was maintained at 30 μL/min.

For Flow-FISH analysis, cells were discriminated from the background by gating on a positive 6-FAM signal for single species analysis. For analyzing the multi-species blend, cells were quantified by gating on a positive signal of the respective fluorescent dye of the specific probe or by gating on 6-FAM to quantify all viable cells. Flow rate was maintained at 30 μL/min.

Experiments to rule out device differences were additionally measured using the CyFlow Cube 6 (Sysmex, Görlitz, Deutschland), with a single 488 nm (50 mW) laser and 5 detectors: forward scatter (FSC), side scatter (SSC) and 3 fluorescence channels (FL1 536/40 nm, FL2 590/50 nm and FL3 RG630 nm). Data analysis was performed using the FCS Express software (*De Novo* Software, FCS Express V5.01.0082).

Quality controls were performed daily before the instruments were used according to the manufacturer’s specifications.

### 2.6 Statistical analysis

Statistical analyses (Dunnett’s test, Wilcox test, R^2^) were performed using RStudio https://www.rstudio.com/ Posit team (2024). RStudio: Integrated Development Environment for R. Posit Software, PBC, Boston, MA. URL http://www.posit.com/.

## 3 Results

### 3.1 Comparison of methods

The accuracy of the Flow-FISH method was evaluated through comparison with Live/Dead (L/D) staining, and conventional plate count for the commonly used probiotic species *Lacticaseibacillus rhamnosus* SP1, *Lactiplantibacillus plantarum* 14D and *Bifidobacterium animalis* subsp. *lactis* BLC1. To compare results, viable cells, active fluorescence units (AFUs) and colony forming units (CFUs) were extrapolated to 1 g of lyophilizate and presented as mean ± standard deviation (SD).

**Figure 2:**
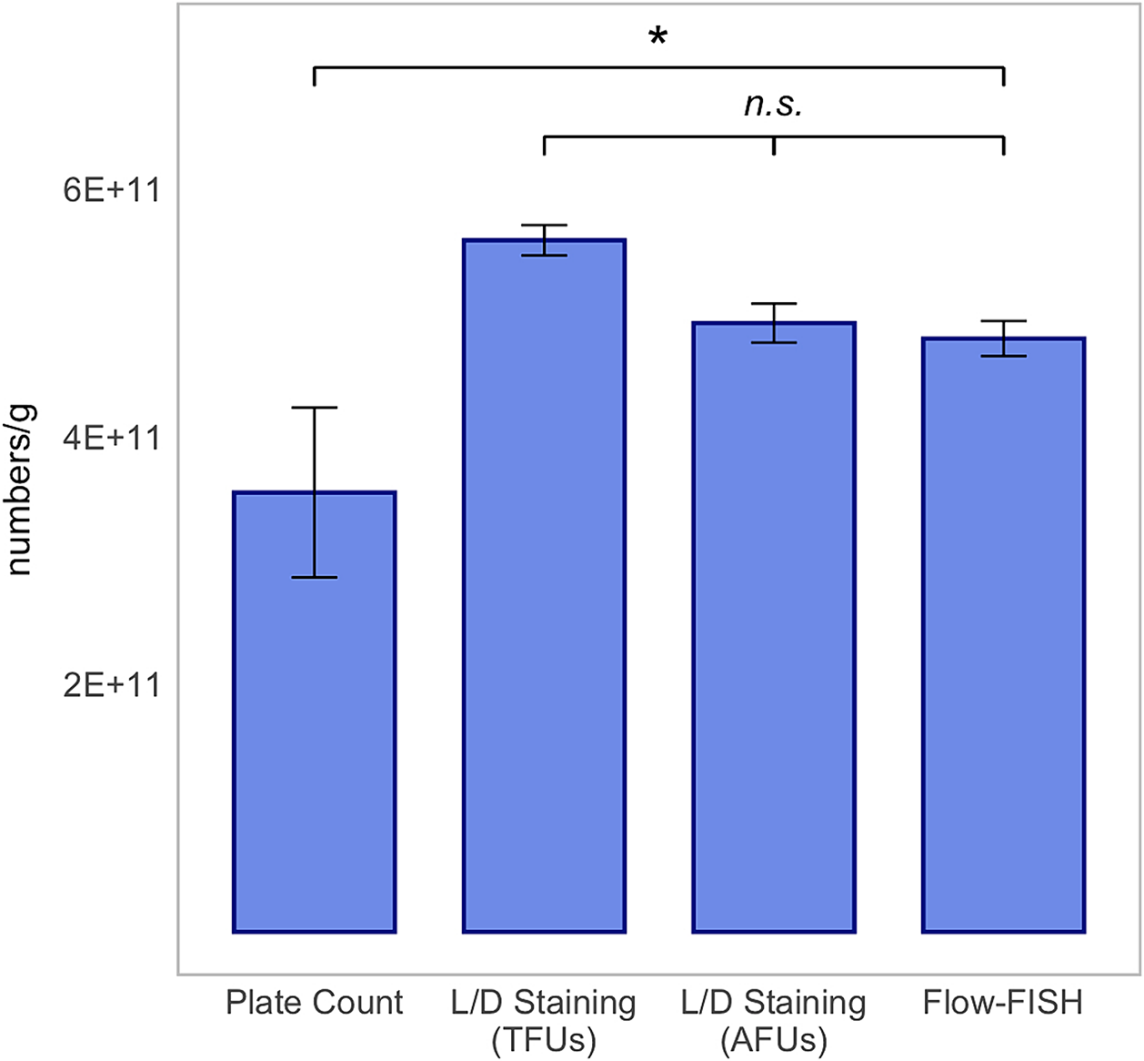
Method comparison for *Lacticaseibacillus rhamnosus* SP1. This analysis includes colony forming units (CFUs) derived from plate count, total fluorescence units (TFUs) and active fluorescence units (AFUs) from L/D staining, and viable cell counts as determined by the Flow-FISH method. Displayed data include the mean values and standard deviations (SDs) for each method. Statistical differences between the Flow-FISH method and the other techniques were assessed using Dunnett’s test (*n* = 3). n.s., not significant, *p* > 0.05 and * = *p* < 0.05.

In our comparison of the Flow-FISH method with L/D staining, we assessed the average of three rehydrated samples, each with five technical replicates, for each organism. For *L. rhamnosus*, the Flow-FISH-detected viable cells numbered at 4.79 × 10^11^ ± 1.42 × 10^10^ per gram and were similar to the count of AFUs detected by L/D staining at 4.92 × 10^11^ ± 1.58 × 10^10^ per gram, and lower than the total fluorescence units (TFUs) count from L/D staining, which was 5.58 × 10^11^ ± 1.23 × 10^10^ (Figure 2).

**Figure 3:**
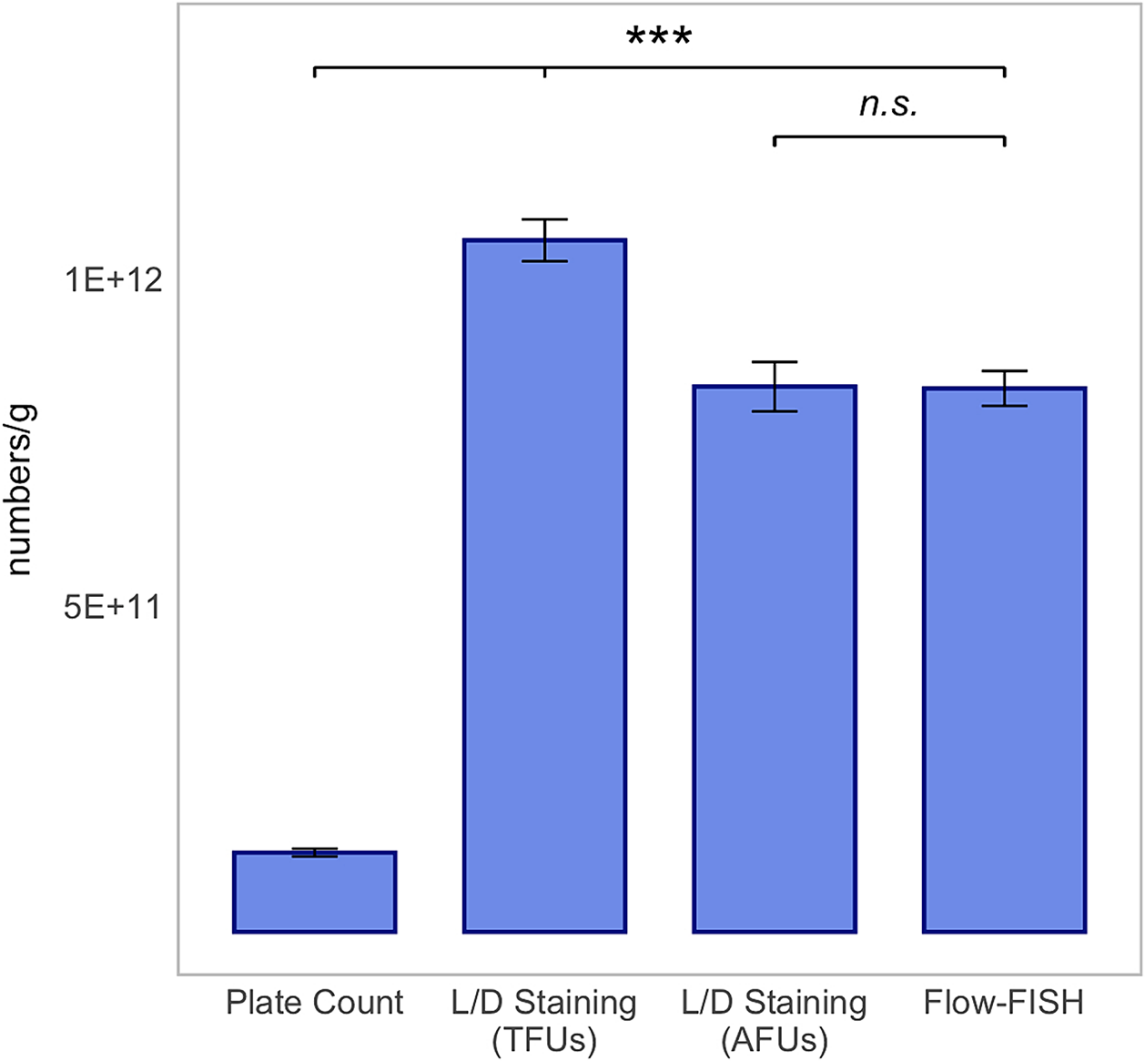
Method comparison for *Lactiplantibacillus plantarum* 14D. This analysis includes colony forming units (CFUs) derived from plate count, total fluorescence units (TFUs), and active fluorescence units (AFUs) from L/D staining, and viable cell counts as determined by the Flow-FISH method. Displayed data include the mean values and Standard Deviations (SDs) for each method. Statistical differences between the Flow-FISH method and the other techniques were assessed using Dunnett’s test (*n* = 3). n.s., not significant, *p* > 0.05, * = *p* < 0.05, ** = *p* < 0.01, and *** = *p* < 0.001.

A similar trend was observed for *L. plantarum* 14D, with the count of viable cells detected by Flow-FISH at 8.32 × 10^11^ ± 2.67 × 10^10^, closely matching the AFUs detected by L/D staining at 8.35 × 10^11^ ± 3.78 × 10^10^, and lower than the TFUs, which were 1.06 × 10^12^ ± 3.21 × 10^10^ (Figure 3).

**Figure 4:**
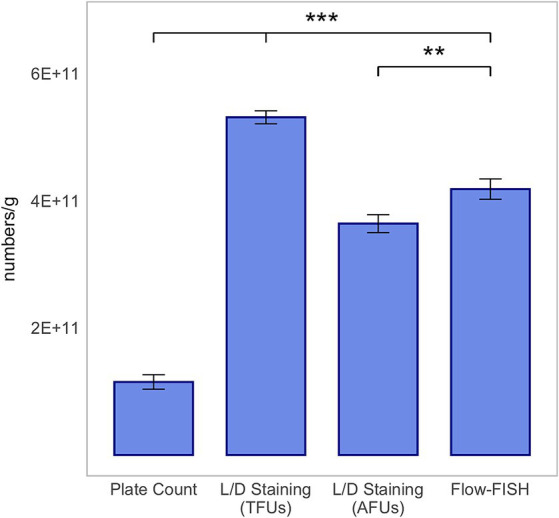
Method comparison for *Bifidobacterium animalis* subsp. *lactis* BLC1. This analysis includes colony forming units (CFUs) derived from plate count, total fluorescence units (TFUs), and active fluorescence units (AFUs) from L/D staining, and viable cell counts as determined by the Flow-FISH method. Displayed data include the mean values and standard deviations (SDs) for each method. Statistical differences between the Flow-FISH method and the other techniques were assessed using Dunnett’s test (*n* = 3). n.s., not significant, *p* > 0.05, * = *p* < 0.05, ** = *p* < 0.01, and *** = *p* < 0.001.

Analysis of *B. lactis* revealed a largely comparable observation, with the exception that the count of viable cells detected by Flow-FISH, at 4.18 × 10^11^ ± 1.60 × 10^10^, was higher than the number of AFUs by L/D staining, which was 3.64 × 10^11^ ± 1.41 × 10^10^, and lower than TFUs of 5.31 × 10^11^ ± 1.02 × 10^10^ (Figure 4).

The CFUs determined by plate count, at 3.55 × 10^11^ ± 6.85 × 10^10^ for *L. rhamnosus*, 1.21 × 10^11^ ± 6.00 × 10^9^ for *L. plantarum* 14D, and 1.15 × 10^11^ ± 1.14 × 10^10^ for *B. lactis*, were lower than those determined by the other two methods. CFUs were determined by the mean value of three rehydrated samples, from which two dilution series were plated.

In summary, the count of viable cells detected by Flow-FISH aligned with the counts determined by L/D staining. Plate count quantified merely 74, 28% or 15% of CFUs compared to Flow-FISH (Figures 2–4).

### 3.2 Precision/repeatability

According to the definition of probiotics as: “live microorganisms that, when administered in adequate amounts, confer a health benefit on the host,” the actual amount of viable cells in the probiotic product is relevant (Hill et al., 2014). Consequently, both manufacturers and consumers need to have a trustworthy method for enumeration that ensures reliable examination of probiotics by demonstrating high repeatability.

To assess the precision, i.e., repeatability, of the different methods, the Flow-FISH and L/D staining procedures were conducted three times with five independently diluted and measured technical replicates. For plate count, two dilution series were prepared, and two different dilutions were plated. The measure of repeatability chosen was the relative standard deviation (RSD).

For Flow-FISH, RSD among technical replicates ranged from 3.13 to 6.91% for *L. rhamnosus*, 2.71 to 6.48% for *L. plantarum* 14D and 5.90 to 6.98% for *B. lactis*. In case of L/D staining, TFUs count showed an RSD from 1.23 to 3.62% for *L. rhamnosus*, 5.14% to 6.80% for *L. plantarum* 14D and 2.79 to 4.69% for *B. lactis*. The RSD for AFUs was between 0.82 and 3.57% for *L. rhamnosus*, 5.83 to 6.48% for *L. plantarum* 14D, and 2.66 and 5.65% for *B. lactis*. The plate count method exhibited quite high RSD values, ranging from 8.56 to 31.07% for *L. rhamnosus*, 4.62 to 17.76% for *L. plantarum* 14D, and 9.97 to 21.97% for *B. lactis*.

In conclusion, the molecular methods, Flow-FISH and L/D staining, demonstrate better repeatability and lower measurement uncertainty compared to the conventional gold standard, the plate count method, within the context of this study.

### 3.3 Linearity

Cell concentrations differ among probiotic products and across various stages of the manufacturing process. Consequently, a method’s capability to accurately analyze different cell concentrations is essential. Accordingly, a linearity analysis of the Flow-FISH method was performed in this study to address this requirement.

Five different concentrations of viable cells per gram were evaluated. The rehydrated *L. rhamnosus*, characterized by a concentration of 4.79 × 10^11^ viable cells per gram as determined through comparative Flow-FISH method experiments previously described, underwent a series of 10-fold serial dilutions in triplicate. This procedure was meticulously conducted until the concentration achieved the theoretical target of 4.79 × 10^7^ viable cells per gram.

Each dilution step was diluted and measured in triplicates, and mean values were used for linearity evaluation.

**Figure 5:**
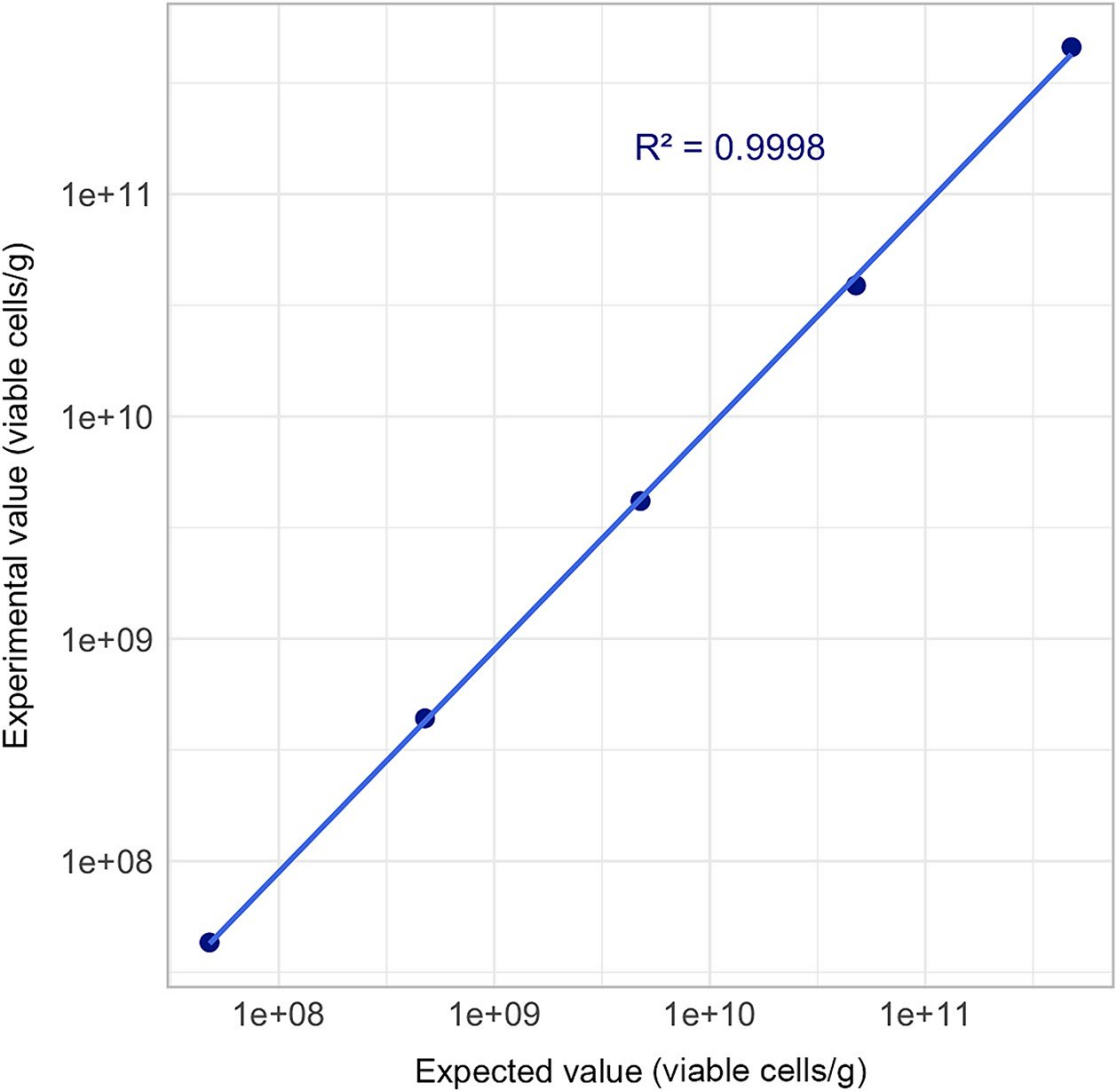
Linearity assessment of Flow-FISH method. Mean quantification results derived from three technical replicates are plotted across a five-point concentration gradient, extending from 4.79 × 10^7^ to 4.79 × 10^11^ viable cells/g. Acceptance limit: *R*^2^ ≥ 0.95.

Linearity was confirmed with an *R*^2^ value of 0.9998 (*R*^2^ ≥ 0.95), thereby validating the Flow-FISH method within a range between 10^7^ and 10^11^ (Figure 5).

### 3.4 Specific enumeration of strains in a multi-species probiotic blend

Probiotic products are typically composed of multiple species, making the Flow-FISH method, with its capability to enumerate each species specifically, highly advantageous. To illustrate the effectiveness of the Flow-FISH method, a mix containing equal amounts of *L. rhamnosus*, *L. plantarum* LP-115, and *B. lactis* lyophilizates was analyzed. Specific oligonucleotide probes were used for each species, each linked to a distinct fluorescent dye (see Materials and Methods). Additionally, the EUB338 probe (Amann et al., 1990b), universally binding to organisms of the kingdom *Bacteria*, enabled the determination of the total viable bacterial count.

Initially, the instruments’ capability to differentiate between the chosen fluorescent dyes was validated through a similarity test.

**Figure 6:**
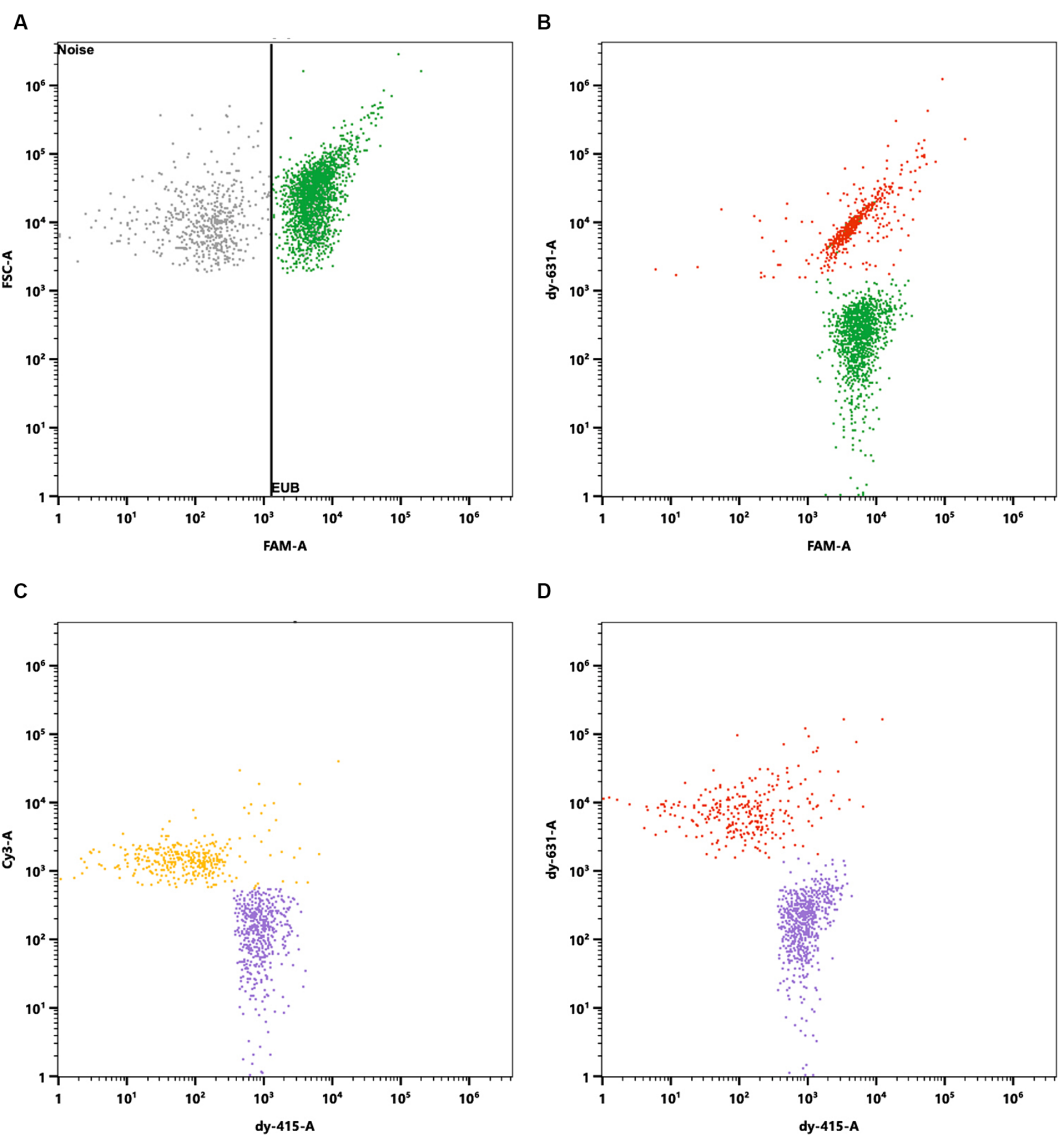
Specific enumeration of bacterial strains in a multi-species probiotic blend via flow cytometry coupled with fluorescence *in situ* hybridization (Flow-FISH). A universal bacterial probe, EUB338 (Amann et al., 1990b), tagged with 6-FAM, facilitated enumeration of total viable bacteria. Specific detection employed probes labeled with distinct fluorophores: *Lacticaseibacillus rhamnosus* SP1 with DY-415, *Lactiplantibacillus plantarum* LP-115 with Cy3, and *Bifidobacterium animalis* subsp. *lactis* with DY-631. **(A)** Signals were plotted against FAM and Forward Scatter (FSC) to distinguish all cells targeted by EUB338 (in green) from background noise. Cells positive for EUB338 were further gated to analyze the distinct strains within the blend. **(B)** FAM signals plotted against DY-631 to separate all cells (green) from *B. lactis* (red), which showed both FAM and DY-631 signals. **(C)** Cy3 signals plotted against DY-415 to distinguish *L. plantarum* (yellow) from *L. rhamnosus* (purple). **(D)** DY-631 signals plotted against DY-415 to differentiate *B. lactis* (red) from *L. rhamnosus* (in purple).

The mixed blend was then processed using the Flow-FISH method and hybridized with a mixture of specific oligonucleotide probes. Each species targeted by a specific probe formed a population of viable cells that could easily be distinguished from background noise and other labeled cells in the sample (Figure 6).

Analysis via the EUB338 probe indicated the presence of 4.54 × 10^11^ viable cells per gram in the mixture. The breakdown of species-specific counts revealed 1.72 × 10^11^ viable cells for *L. rhamnosus*, 1.25 × 10^11^ viable cells for *B. lactis*, and 1.83 × 10^11^ viable cells for *L. plantarum* LP-115 per gram. The discrepancy of 5.31% between the total cell count and the cumulative species-specific count is attributed to the methodological uncertainty, validating the species-specific detection capability of Flow-FISH.

### 3.5 Robustness: device comparison

For the validation of the Flow-FISH method destined for quantification of probiotic products, robustness, particularly its independence from specific equipment, is necessary.

Therefore, five technical replicates of rehydrated *L. rhamnosus* were measured using two different flow cytometers: Cytek Northern Lights and Sysmex CyFlow Cube 6. Results are presented as mean ± SD.

**Figure 7:**
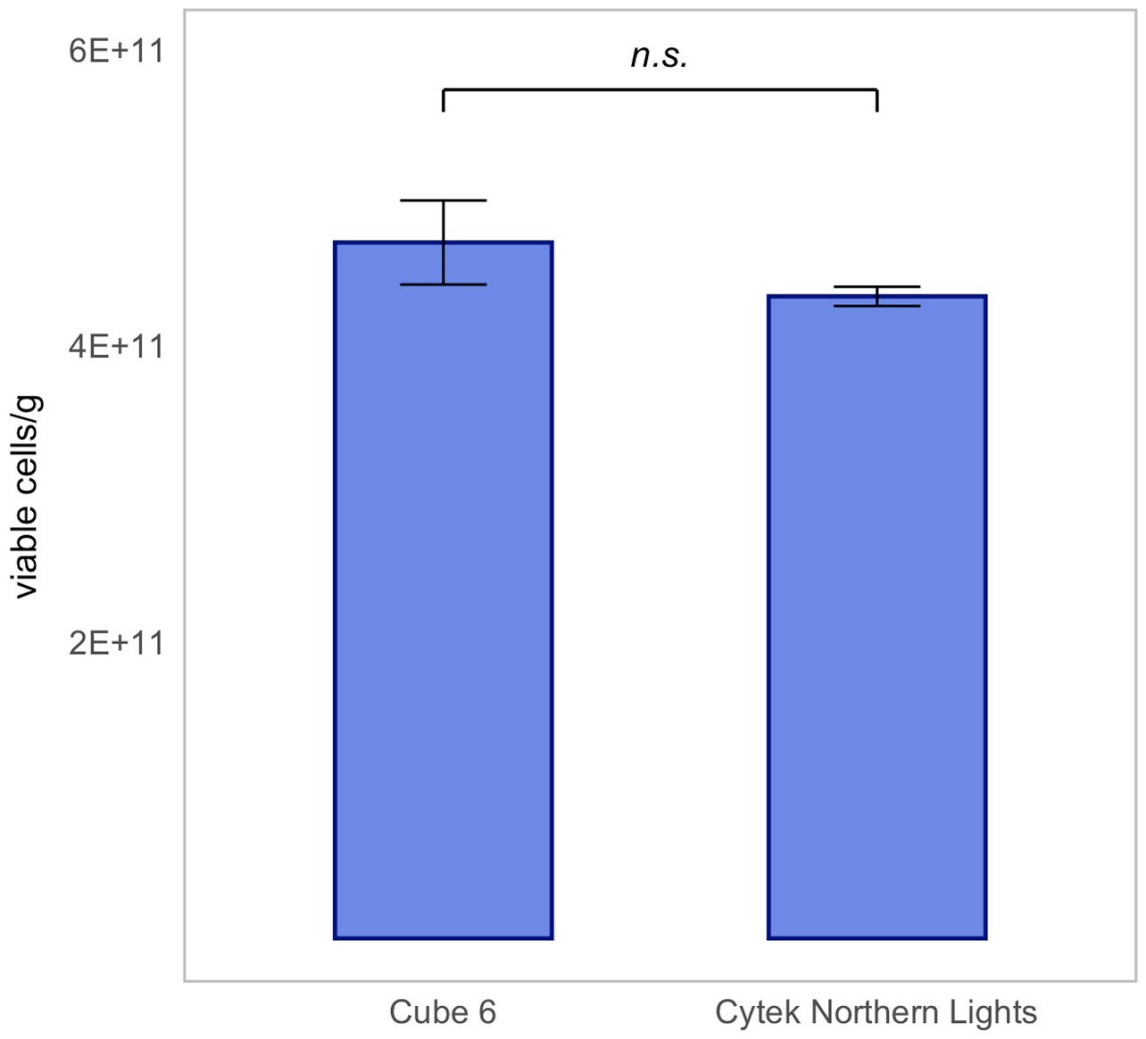
Device-independent validation of Flow-FISH measurements for *Lacticaseibacillus rhamnosus* SP1. Results obtained from lyophilized bacterial samples quantified on two different flow cytometers, illustrating the method’s consistency across instruments, were compared. Presented are the mean values and their corresponding standard deviations (SD). The Wilcoxon test was applied to assess statistical differences between the devices (*n* = 5). n.s., not significant, *p* > 0.05.

The same dilution was measured on each instrument. With the Cytek Northern Light measuring an extrapolated average of 4.36 × 10^11^ ± 1.38 × 10^10^ viable cells/g and the Sysmex CyFlow Cube 6 measuring 4.60 × 10^11^ ± 2.54 × 10^10^ viable cells/g, there was no significant difference between the results of the two instruments (Figure 7). This proves that the Flow-FISH method is not dependent on the device used.

### 3.6 Analysis of a commercial end product

**Figure 8:**
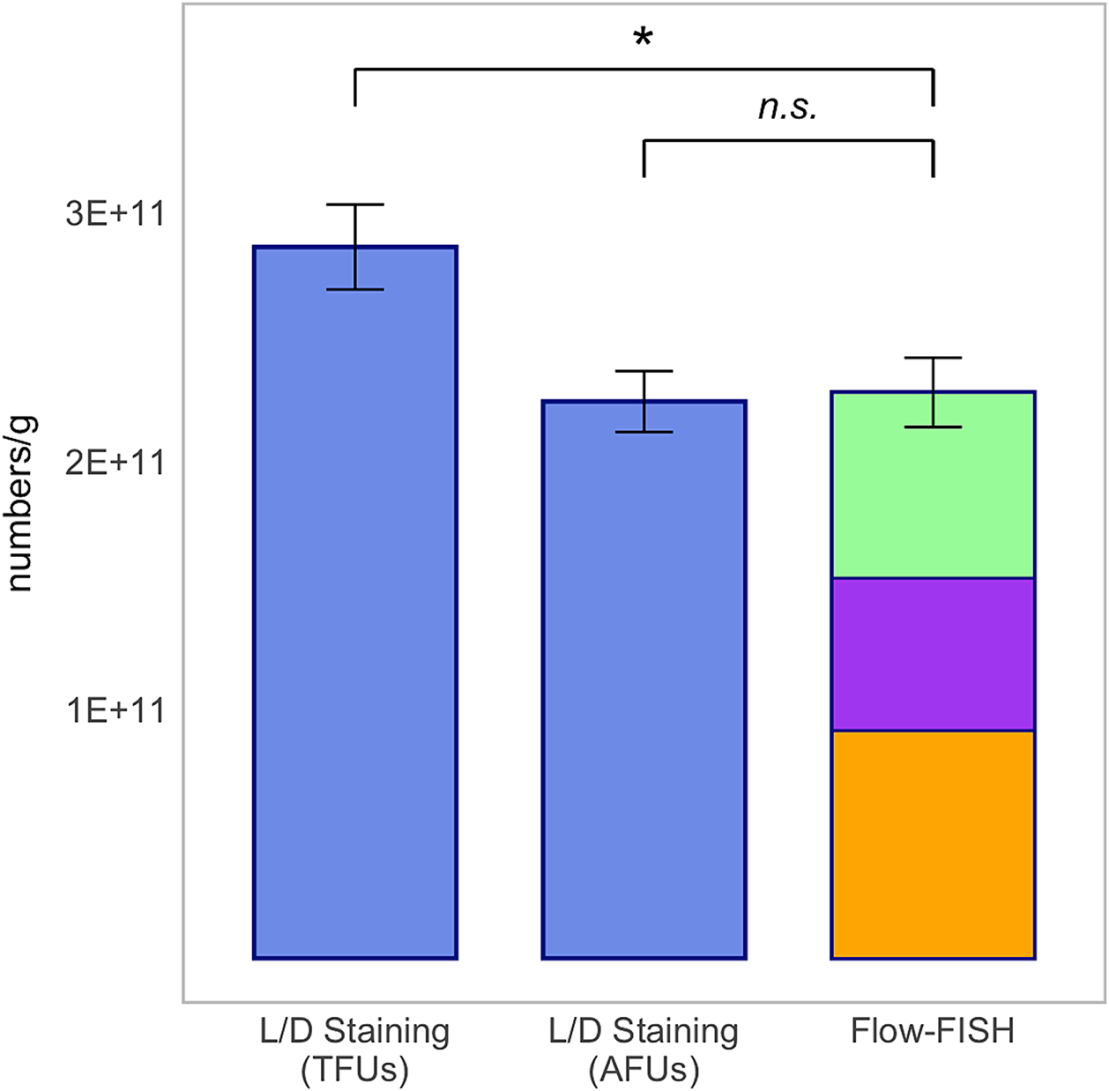
Method comparison for the commercial product “IberoBiotics Pro” (Lot number: 69974, expiry date: February 2025; Bayer Vital GmbH, Leverkusen, Germany). This analysis includes total fluorescence units (TFUs) and active fluorescence units (AFUs) derived from L/D staining, and viable cell counts as determined by the Flow-FISH method. Displayed data include the mean values and standard deviations (SDs) for each method. The Flow-FISH bar is color-coded to represent the mean values of individual species detected: *Lacticaseibacillus rhamnosus* in orange, *Lactobacillus acidophilus* in purple, and *Bifidobacterium animalis* subsp. *lactis* in green. Statistical differences between the Flow-FISH method and the other techniques were assessed using Dunnett’s test (*n* = 3). n.s., not significant, *p* > 0.05 and * = *p* < 0.05.

Since bacterial strains undergo often various stressors during their processing into probiotic end products (Fenster et al., 2019; Kiepś and Dembczyński, 2022), it was necessary to demonstrate that the Flow-FISH protocol is also effective when analyzing a commercial end product. Therefore, the “IberoBiotics Pro” (Bayer Vital GmbH, Leverkusen, Germany) was analyzed using the Flow-FISH method and L/D staining for comparison. According to the manufacturer, it contains 6 × 10^9^ CFUs per capsule, with each capsule containing approximately 300 mg of powder. The viable cells determined by Flow-FISH were 2.28 × 10^11^ ± 1.39 × 10^10^ per gram, which was comparable to the AFUs detected by L/D staining at 2.24 × 10^11^ ± 1.22 × 10^10^ per gram. The TFUs were higher, with a total count of 2.86 × 10^11^ ± 1.70 × 10^10^ per gram. In detail, the viable cell count determined by Flow-FISH for *L. rhamnosus* was 9.16 × 10^10^ ± 6.80 × 10^9^, for *L. acidophilus* was 6.13 × 10^10^ ± 4.35 × 10^9^, and for *B. lactis* was 7.46 × 10^10^ ± 3.99 × 10^9^ per gram (Figure 8).

## 4 Discussion

In this study, it was demonstrated that the optimized Flow-FISH method for gram-positive probiotic species represents a rapid, robust, and easily implementable technique. We compared the optimized Flow-FISH method with Live/Dead (L/D) flow cytometry and with the conventional plate count technology.

The Flow-FISH method showed that it yields results comparable to those of the established L/D staining but is additionally capable of specifically quantifying viable single species in multi-species blends. Compared to the gold standard plate count, the Flow-FISH method and likewise the L/D staining flow cytometry revealed higher and more precise results.

### 4.1 Comparison Flow-FISH vs. Live/Dead staining

An established method for enumerating probiotics is the L/D staining. It differentiates living from dead cells based on the integrity of the cell wall, performing under the assumption that non-viable cells lack an intact one. Fluorescent nucleic acid dyes are used, which have different capabilities for cell penetration depending on membrane integrity (Díaz et al., 2010; ISO 19344:2015). In contrast to the plate count method, it is significantly faster and easier to execute, as it does not require consideration of the various growth conditions of the cells. Moreover, cells are analyzed based on the criterion of cell permeability rather than their ability to grow, which leads to a more accurate count of viable cells (Lahtinen et al., 2005; Foglia et al., 2020; Visciglia et al., 2022). However, strict adherence to the staining times of the cells with the dye requires effective time management in the laboratory. This may result in periods of personal inactivity because the precision required during these stages prevents the concurrent performance of other tasks.

In our study, L/D staining effectively quantified living cells, yielding counts similar to those obtained via the Flow-FISH method. This congruence occurred despite the distinct assumptions underlying each technique regarding cell viability. Our results are in accordance with those of Lahtinen et al. (2008), who demonstrated similar findings when comparing cell membrane permeability to 16S rRNA content.

Given that probiotic cells are not only exposed to lyophilization as a stressor but also to thermal, osmotic, and oxidative stress factors, this may lead to variations in lysozyme stability and increased susceptibility (Fenster et al., 2019; Kiepś and Dembczyński, 2022). In this study, we demonstrated that the advanced Flow-FISH protocol is applicable not only to lyophilized probiotic gram-positive bacteria but also to a commercial end product, showing a comparable number of viable cells according to Flow-FISH and AFUs as determined by L/D staining, thereby confirming the reliability of the method without requiring adjustments in lysozyme concentration.

Flow-FISH, similar to L/D staining, can also determine the ratio of live to dead cells by employing a cell wall-permeable DNA dye that penetrates all cells, irrespective of their viability status. This approach enables the quantification of living cells relative to the total cell count, offering a comprehensive assessment of cell viability within a sample.

### 4.2 Comparison Flow-FISH vs. plate count

FISH accurately detects cells by using fluorescent probes that bind to specific rRNA sequences, forming stable RNA–DNA hybrids (Amann et al., 1990a). This method’s sensitivity is greatly enhanced by the high number of rRNA molecules present in viable cells, which ranges from a few hundred to 100,000 per cell, integral to ribosomal function (Amann and Fuchs, 2008). The abundant rRNA, when labeled with these probes, produces a strong cumulative fluorescence signal upon excitation with high-energy light. This signal is detectable, enabling precise cell identification and localization based on rRNA expression (Amann et al., 1990a).

In contrast, plate count measures colony forming units (CFUs). These numbers are often underestimated because the indirect nature of the method does not guarantee that a colony derives from one single cell (Davis, 2014; Pereira et al., 2023). Moreover, in blends, which represent the majority of probiotic products, different species may inhibit each other and thus interfere with growth, leading to an underestimation and not reflecting the actual quantitative composition of the product (Avonts et al., 2004; Sielatycka et al., 2021).

Additionally, the plate count method is impractical for manufacturers’ quality control, as it can involve long incubation steps that extend over several days. Also, plate count is laborious since it cannot be uniformly applied to all probiotic organisms, as distinct growth conditions, such as optimal temperatures, atmospheric oxygen levels, and particular nutrients are required for different species. Moreover, optimal growth conditions remain unknown for many microorganisms. Furthermore, this methodology lacks the ability to differentiate between closely related organisms and there is no guarantee that a colony results from a single cell rather than from an aggregate or chain of cells (Lahtinen et al., 2006a; Davis, 2014; Jackson et al., 2019; Vinderola et al., 2019).

Moreover, due to stress during manufacturing and storage, cells in probiotic products often enter the so-called viable but non-culturable (VBNC) state, where they are metabolically active and contain still high levels of rRNA, but might be not capable of replication (Bao et al., 2023). Nevertheless, they may have probiotic properties (Breeuwer and Abee, 2000; Lahtinen et al., 2006b, 2008; Fiore et al., 2020; Wendel, 2022).

For these reasons, plate count is nowadays considered more of an estimate than an actual quantification of viable cells (Davey, 2011; Boyte et al., 2023).

Therefore, the combination of flow cytometry with FISH, the so-called Flow-FISH method, offers a faster, more accurate and specific alternative. The results of this study, show more viable cells detected by Flow-FISH as well as with L/D staining compared to CFUs, indicate that Flow-FISH offers a more accurate representation compared to plate count. This is further supported by the fact that the Flow-FISH method is significantly more reproducible than plate count, as could be shown by our data, highlighting the issues with cultivation methods.

### 4.3 Advanced Flow-FISH protocol

The Flow-FISH protocol developed in this study presents significant advantages over existing protocols for analyzing gram-positive bacteria, addressing a longstanding challenge in the field. The concept of merging flow cytometry and FISH originated around three decades ago (Amann et al., 1990a; Wallner et al., 1993; Snaidr et al., 1999), and various probiotic and fecal samples have been analyzed using this combination (Rigottier-Gois et al., 2003; Rochet et al., 2004; Vaahtovuo et al., 2005; Dinoto et al., 2006; Collado and Sanz, 2007; Cleusix et al., 2010). However, such studies often proved to be challenging, especially because probiotics are typically lactic acid bacteria, which are gram-positive (Williams, 2010). The complexity arises from their cell wall structure, particularly the thicker peptidoglycan layer, which impedes the penetration of labeled oligonucleotide probes (Chapot-Chartier and Kulakauskas, 2014). Enzymatic treatments are mostly used to overcome these challenges and enable the effective diffusion of the probes into ethanol or paraformaldehyde fixed cells (Beimfohr et al., 1993). In our protocol, we combined highly concentrated lysozyme treatment of unfixed cells with a quenching step.

Therefore, previous FISH/flow cytometric protocols were not only time-consuming, requiring hybridization times of more than ten hours, but also labour-intensive because of necessary centrifugation steps.

The protocol optimized in this study has significantly reduced the hybridization time to just 1.5 h, resulting in a total protocol duration of merely 2 to 2.5 h. At the same time, it decreased the workload and minimized the risk of cell loss by eliminating the need for centrifugation steps. The required signal-to-noise ratio was achieved through a final dilution series and the inclusion of complementary quenchers in the washing buffer. These quenchers, designed to be complementary to the oligonucleotide probes, bind to any unbound probes, effectively suppressing their free signal and enhancing the specificity and clarity of the detection (Beimfohr et al., 2010).

In summary, the optimized Flow-FISH method protocol is effective for the reliable enumeration of probiotics, yielding results comparable in quantification and precision to the established L/D staining method. Moreover, due to its optimization, the protocol is user-friendly, necessitating minimal handling time, with a total duration, including incubation steps, of 2 to a maximum of 2.5 h.

In addition to its speed and practicality, the Flow-FISH method is also a robust technique. This study demonstrated that there is no significant difference in the results when technical replicates of the same sample are measured on different devices, indicating that the method’s accuracy and reliability are independent of the specific device used. Furthermore, it was shown that cells can be reliably quantified across a wide range, from approximately 5 × 10^7^ to 5 × 10^11^ cells per gram, demonstrating the method’s broad applicability for analyzing samples with vastly different cell densities. Given that this range of cell counts is typical in both the manufacturing process and the final probiotic product, the Flow-FISH method proves suitable for quality control at in-process and end-process stages. This adaptability ensures accurate monitoring and validation of probiotic concentrations, which is crucial for maintaining product efficacy and regulatory compliance. Moreover, unlike L/D staining, the Flow-FISH method does not require immediate measurement of samples after staining. Our findings indicate that a delay between staining and measurement does not alter the results (data not shown), providing greater flexibility in daily laboratory operations. This characteristic enhances workflow efficiency, allowing for better planning and resource allocation without compromising the accuracy of the quantification.

Recently, there has been an increase in the prevalence of probiotic products containing spore-forming bacteria (Elshaghabee et al., 2017). However, since the majority of probiotic products on the market are composed of lactic acid bacteria, which are not spore formers (Chapot-Chartier and Kulakauskas, 2014), the protocol is optimized for lyophilized products without special consideration for spores. Spore-forming bacteria are particularly resilient due to their ability to form endospores (Elisashvili et al., 2019). Endospores possess a thick cell wall, composed of multiple layers including the cortex and spore coat, which provides substantial protection against environmental stresses but also makes them less accessible to FISH oligonucleotide probes (Filion et al., 2009). According to Chambon et al. (1968), endospores contain comparable rRNA content to vegetative cells, and Filion et al. (2009) successfully stained *Bacillus* spores with FISH using an optimized procedure to effectively penetrate the spores. For the specific detection of spores, the Flow-FISH protocol would require modifications. As spore detection was not within the scope of this study the Flow-FISH protocol described is optimized for the current market situation. It should also be noted that the Flow-FISH method was specifically developed for the reliable enumeration of probiotic viable cells and is not intended for the identification or control of contaminants.

In summary, our refined Flow-FISH protocol offers significant improvements over earlier methods that integrate FISH with flow cytometry. We chose not to use ethanol or paraformaldehyde for bacterial fixation (Manz et al., 1992), achieving cell wall permeability but high lysozyme and fluorescently labeled oligonucleotide probes concentrations instead. Through meticulous dilution of the hybridized sample and the use of quencher probes, our approach ensures precise and reliable outcomes. By reassessing the foundational principles of FISH, we have developed a method that enables the robust, rapid and specific identification of gram-positive probiotic bacteria of the genera *Lactobacillus* and *Bifidobacterium* through a synergistic combination with flow cytometry.

### 4.4 Analysis of multi-species probiotic blends

Probiotic products typically contain a mixture of different species, each with varying survival rates within lyophilized products (Drago et al., 2004). Given this variability, it is inadequate to merely calculate the initially added proportion of each species relative to the total number of living cells. A significant advantage of the Flow-FISH method over L/D staining and the plate count method lies in its specificity (Jackson et al., 2002). This specificity is crucial for accurately quantifying the individual species within a probiotic blend, ensuring the product’s efficacy. Flow-FISH combines this specificity with the measurement of viable cells, offering a distinct advantage in the precise quantification of individual species within probiotic blends.

In the experiment conducted in this study, which aimed at specific enumeration within a multi-species blend, it was demonstrated that the Flow-FISH method is capable of identifying not only the total count of living bacterial cells but also the proportions of the three different species, *L. rhamnosus*, *L. plantarum*, and *B. lactis*.

Using different fluorescent labels linked to the specific oligonucleotides, the three species, as well as the total population of all living cells, could be detected within a single hybridization and measurement step.

This method enables a highly efficient process, where just a single analysis of the sample is sufficient to determine the total count of all living cells, their percentage share, and the precise quantification of each individual species. Given that gram-positive bacteria do not all require the same lysozyme treatment for effective probe penetration (Beimfohr et al., 1993), variations in the species blend may necessitate conducting more than one analysis. By categorizing species according to their similar treatment needs, a comprehensive evaluation of the probiotic blend can be achieved. This approach ensures that the unique cell wall characteristics of different gram-positive species are adequately addressed, allowing for accurate and effective quantification of each species within the blend, as shown by the analysis of the commercial product. Due to the specificity of the oligonucleotide probes, accurate quantification also remains feasible when different analytical approaches are used on the same sample. This highlights the method’s utility in accurately quantifying complex probiotic formulations.

### 4.5 Summary and outlook

This study has shown that the Flow-FISH method, refined with our protocol, excels in analyzing probiotic products. It outperforms both the L/D staining and traditional plate count methods by offering the combination of rapidity and specificity together with robustness and a better suitability for laboratory workflows.

Its proficiency in evaluating additional probiotic blends further establishes its utility for comprehensive quality control, making it an invaluable asset for both in-process and final product assessments, thereby ensuring product quality and efficacy.

## Data availability statement

The original contributions presented in the study are included in the article/supplementary material, further inquiries can be directed to the corresponding author.

## Author contributions

LS: Writing – review & editing, Writing – original draft, Validation, Project administration, Methodology, Investigation, Formal analysis, Data curation, Conceptualization. PM: Writing – review & editing, Methodology, Investigation, Data curation, Conceptualization. CB: Writing – review & editing, Supervision, Conceptualization. CK: Writing – review & editing, Methodology. CR: Writing – review & editing. JS: Writing – review & editing, Supervision, Conceptualization.
